# Supplementary material for: An efficient and multiple target transgenic RNAi technique with low toxicity in Drosophila
Source: Nat Commun. 2018 Oct 8;9:4160. doi: 10.1038/s41467-018-06537-y (PMC6175926; doi:10.1038/s41467-018-06537-y)
Supplement: Supplementary file 1 — Supplementary Information [file 41467_2018_6537_MOESM1_ESM.pdf]

Supplementary Information

An efficient and multiple target transgenic RNAi technique with low toxicity in *Drosophila*

Qiao et. al.

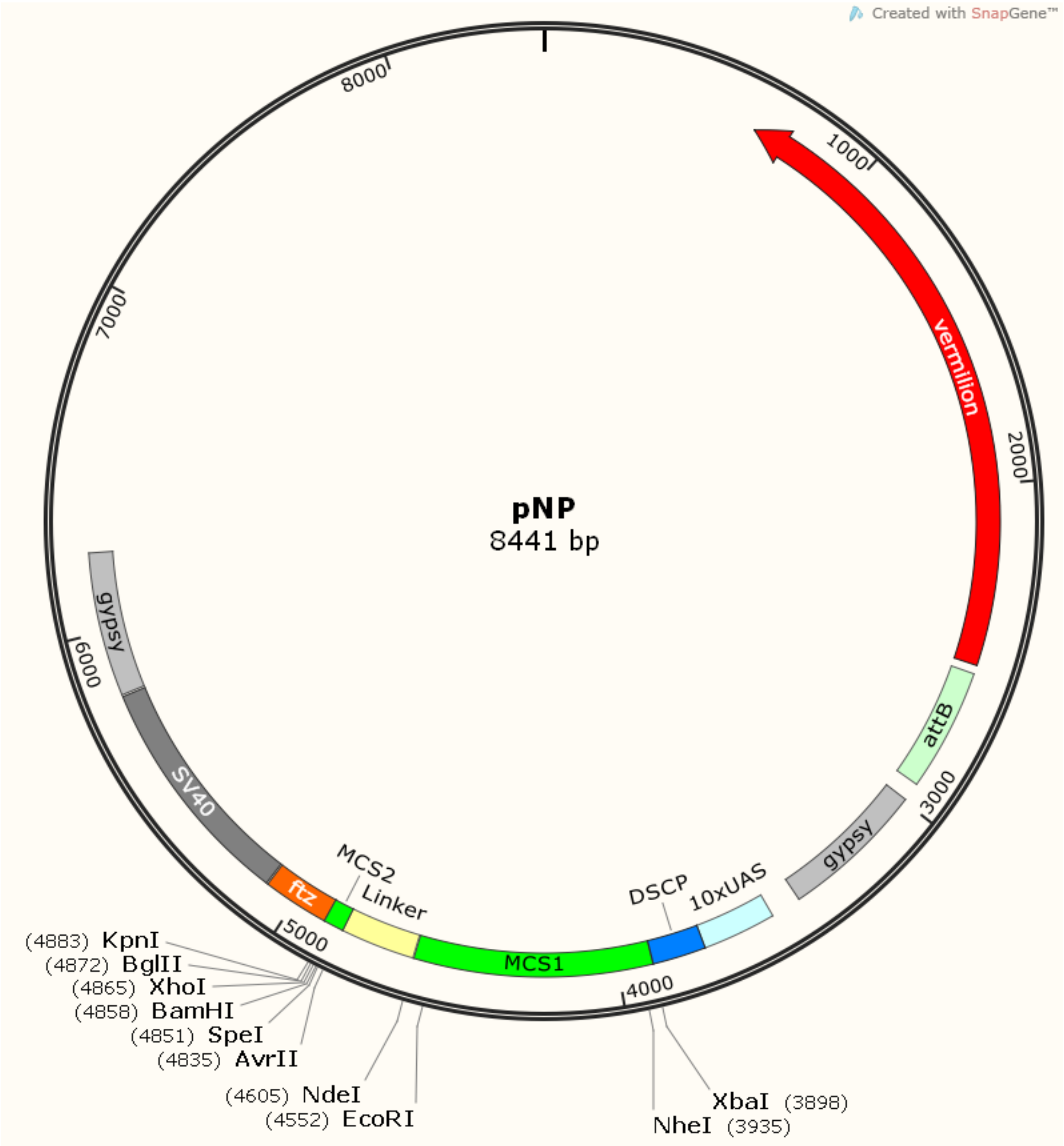

Supplementary Figure 1

Map of pNP.

The pNP vector contains *vermilion* as a selectable marker, and an attB sequence to allow for phiC31 targeted integration at genomic attP landing sites. The *Drosophila* synthetic core promoter (DSCP) contains the TATA, Inr, MTE, and DPE sequence motifs. An intergenic linker between miR-2a-1 and miR-2b-2 allows precise processing and expression of multiple shRNAs. MCS1 allows a single shRNA to be cloned in both orientations, while with the help of MCS2 and the linker it could simultaneously generate multiple shRNAs once. The ftz intron, followed by the SV40 poly(A) tail, facilitates hairpin RNA expression, processing and export from the nucleus. The relevant sequences are flanked by two gypsy insulators to ensure stable transgenic expression.

a

Symbol	Phenotypes	Symbol	Phenotypes
U2af50	partial lethal	CG7006	pupal lethal
SmG	lethal	CG5808	lethal
eIF-2alpha	pupal lethal	Spx	lethal
unk	pupal lethal	SmB	lethal
elav	lethal	mxc	lethal

b

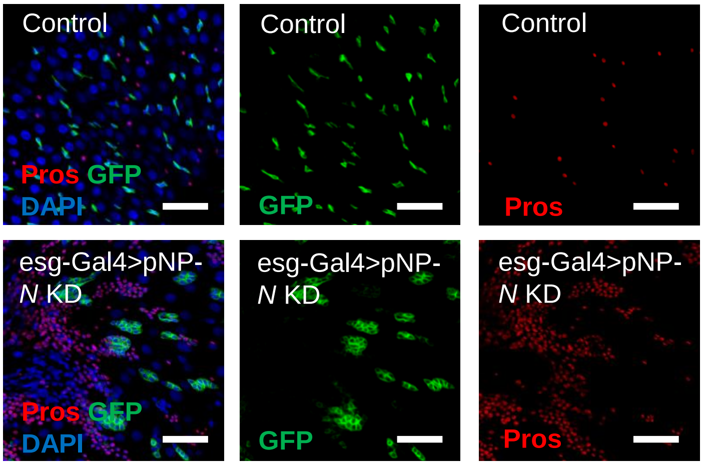

c **d**

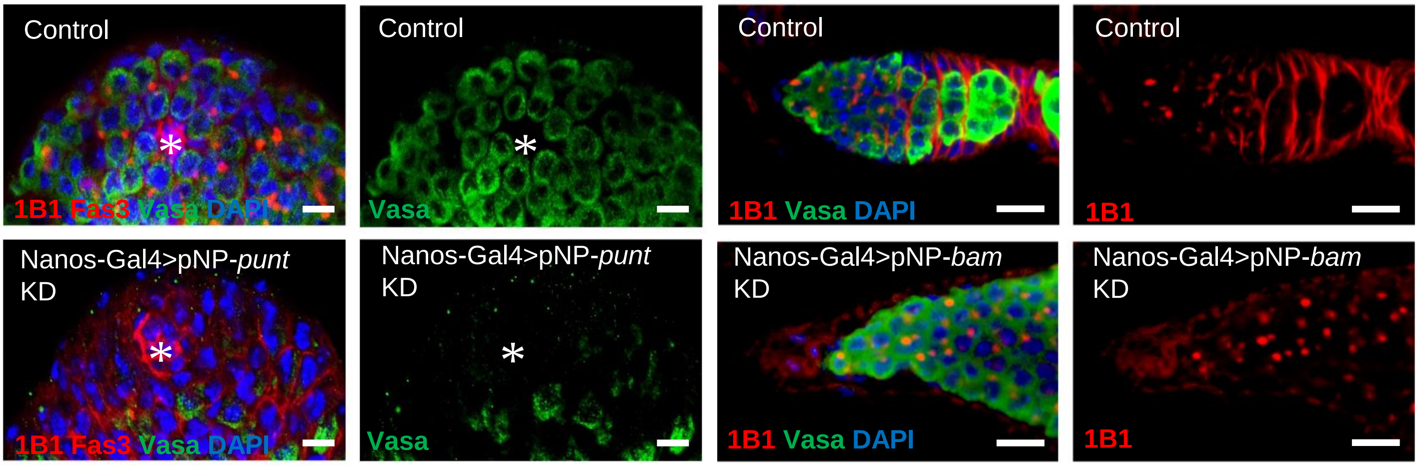

Supplementary Figure 2

The pNP system works efficiently in the neurons, intestine, testis and ovarian stem cell system.

(a) Phenotypes of flies when target genes were knocked down in the neurons system driven by elav-Gal4. (b) Knockdown of *Notch* in the gut driven by esg-Gal4, UAS-*GFP*, tub-Gal80^{ts}/Cyo increased the number of intestinal stem cells (ISCs) and eneroendocrine cells (EE cells) compared with control. GFP (green) marks ISCs, Pros (red) marks EE cells, and DAPI (blue) marks nuclei. Scale bars, 20 μm. (c) The tip of a wildtype testis showing seven germline stem cells (GSCs) that contact the hub cells marked by asterisk, while the tips of *punt* knockdown testis showing no GSCs and germ cells close to the hub cells. Vasa (green) marks germ cells, Fasciclin III (red) marks the hub (asterisk), 1B1 (red) marks spectrosomes/fusomes, and DAPI (blue) marks nuclei. Scale bars, 10 μm. (d) Immunostaining of wild type ovary showing two GSCs in the germrium, while knockdown of *bam* increased the number of GSCs. Vasa (green) marks germ cells, 1B1 (red) marks spectrosomes/fusomes, and DAPI (blue) marks nuclei. Scale bars, 10 μm.

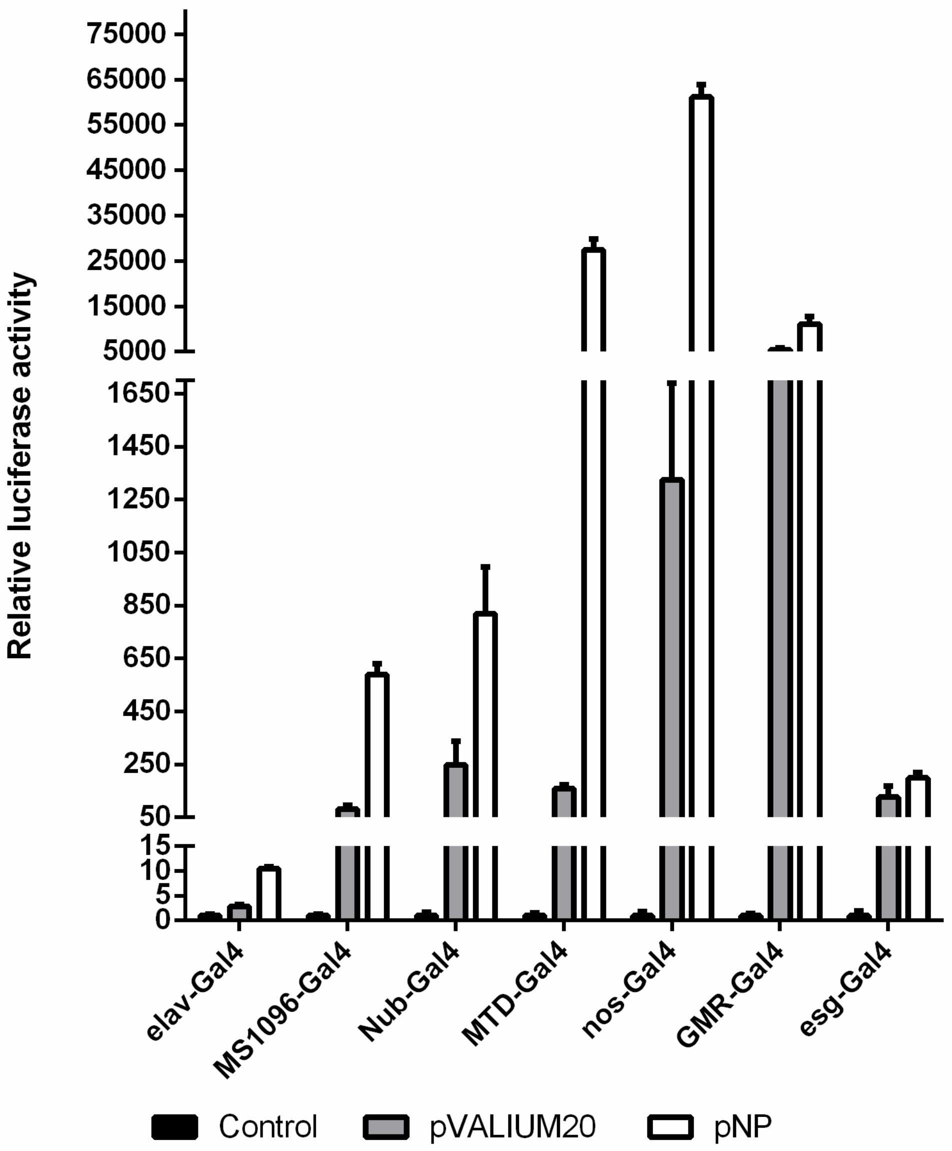

Supplementary Figure 3

The luciferase levels were significantly higher in pNP system than the pVALIUM20 system driven by different Gal4 lines.

Relative luciferase expression levels at different tissues of pVALIUM20-*luciferase* and pNP-*luciferase* flies with different Gal4 drivers (n=5, mean±s.d.).

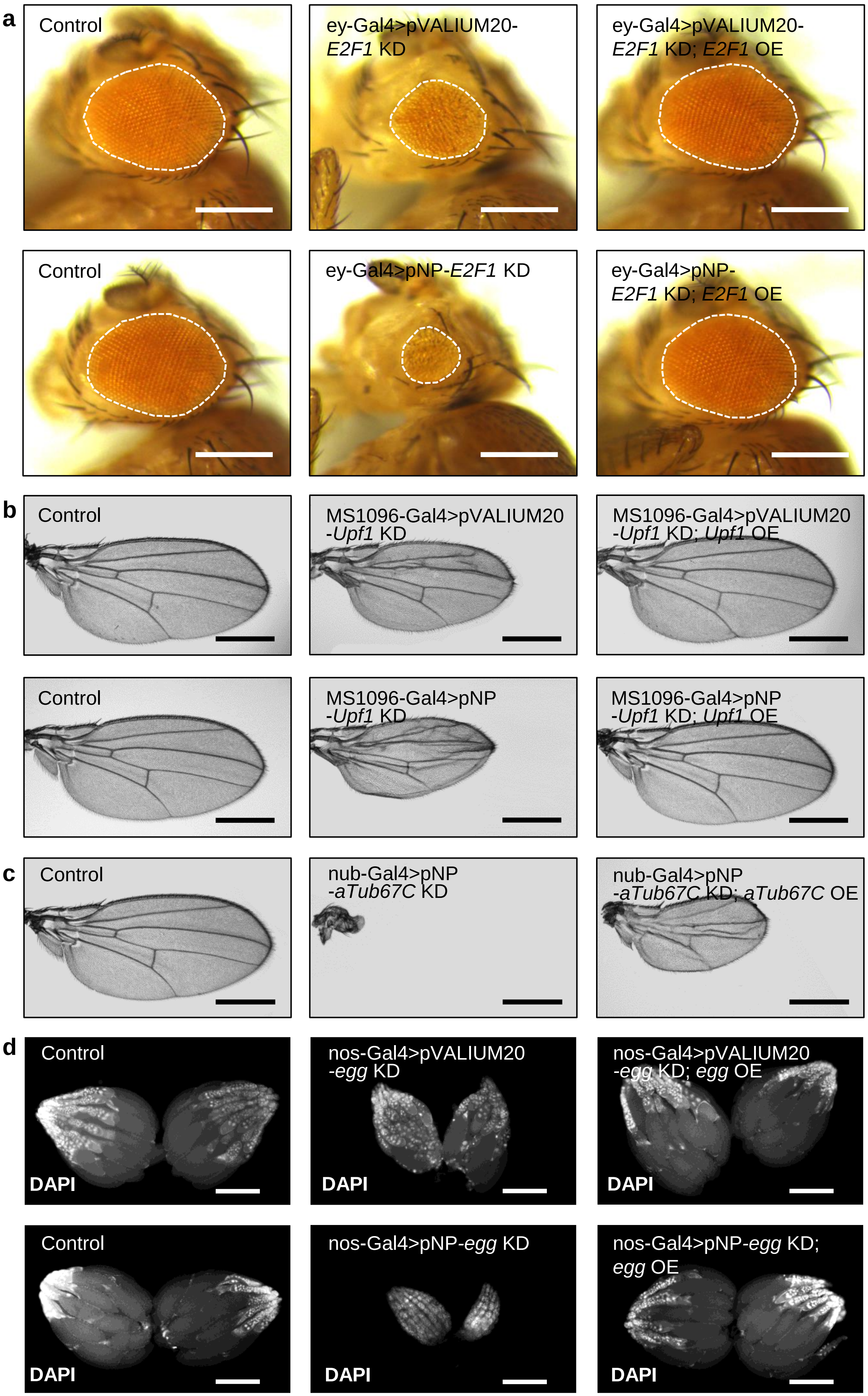
a
Control
ey-Gal4>pVALIUM20-
E2F1 KD
ey-Gal4>pVALIUM20-
E2F1 KD; *E2F1* OE
Control
ey-Gal4>pNP-*E2F1* KD
ey-Gal4>pNP-
E2F1 KD; *E2F1* OE
b
Control
MS1096-Gal4>pVALIUM20
-*Upf1* KD
MS1096-Gal4>pVALIUM20
-*Upf1* KD; *Upf1* OE
Control
MS1096-Gal4>pNP
-*Upf1* KD
MS1096-Gal4>pNP
-*Upf1* KD; *Upf1* OE
c
Control
nub-Gal4>pNP
-*aTub67C* KD
nub-Gal4>pNP
-*aTub67C* KD; *aTub67C* OE
d
Control
DAPI
nos-Gal4>pVALIUM20
-*egg* KD
DAPI
nos-Gal4>pVALIUM20
-*egg* KD; *egg* OE
DAPI
Control
DAPI
nos-Gal4>pNP-*egg* KD
DAPI
nos-Gal4>pNP-*egg* KD;
egg OE
DAPI

Supplementary Figure 4

The phenotypes produced by pNP system and pVALIUM20 system can be significantly rescued by co-expression of target genes.

(a) Knockdown of *E2F1* in the eye using the pVALIUM20 system or the pNP system with the same shRNA driven by ey-Gal4 can be rescued by *E2F1* overexpression, respectively. Scale bars, 200 μm. (b) RNAi of *Upf1* by the pVALIUM20 system or the pNP system in the wing using MS1096-Gal4 can be rescued by *Upf1* overexpression, respectively. Scale bars,500 μm. (c) Targeting of *aTub67C* with the pNP system driven by Nub-Gal4 can be rescued by *aTub67C* overexpression. We did not performed this rescue assay in pVALIUM20- *aTub67C* flies, as it showed no phenotype driven by Nub-Gal4. Scale bars, 500 μm. (d) Knockdown of *egg* in the germline controlled by nos-Gal4 can be rescued by *egg* overexpression. Scale bars, 300 μm.

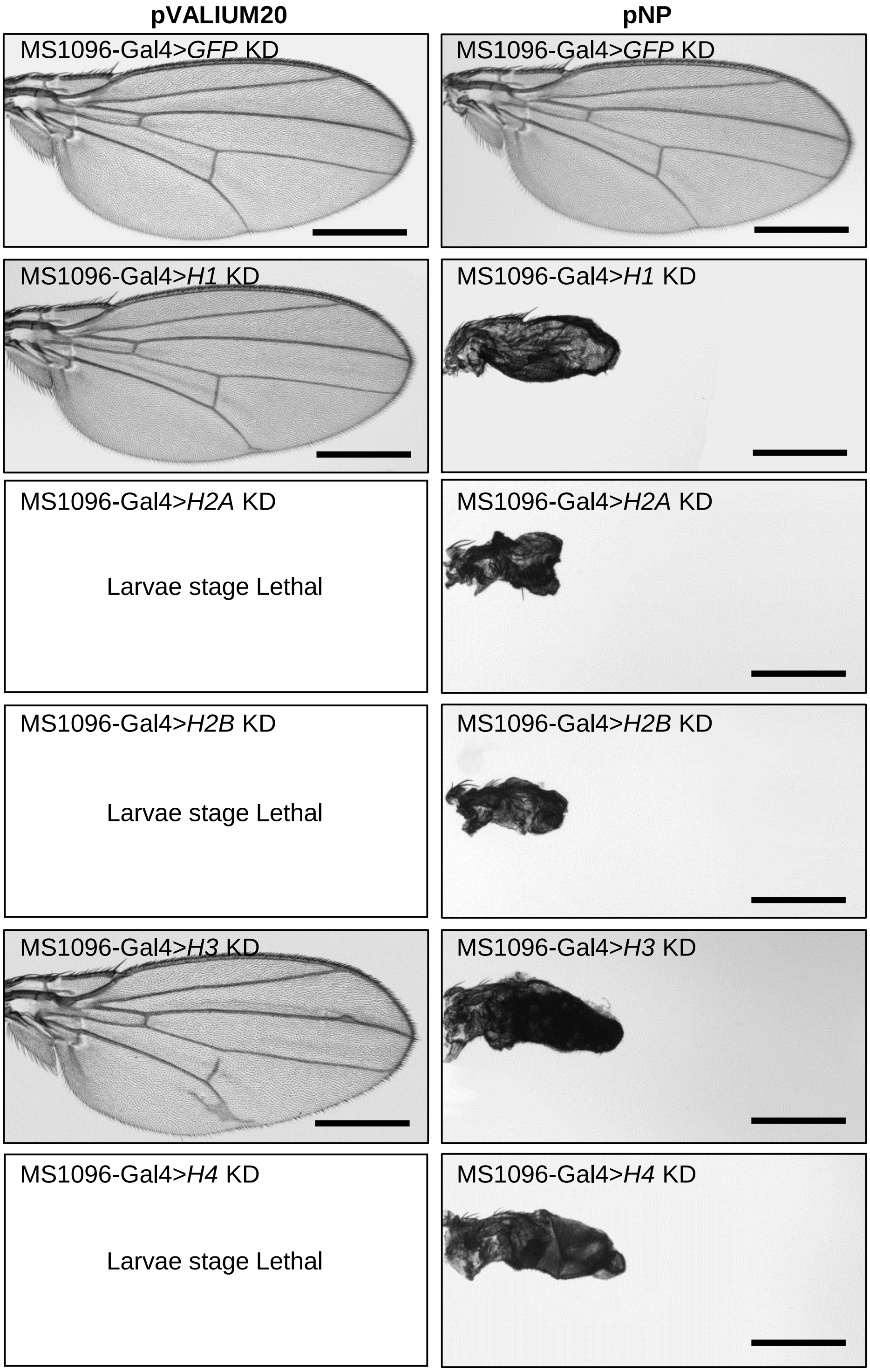

Supplementary Figure 5

The pNP system could efficiently modulate high expression genes.

When driven by MS1096-Gal4, knockdown of all the histones using the pNP system generated a phenotype with severe wing developmental defects. Using the pVALIUM20 system, depletion of H1 and H3 produced an almost wild type phenotype, while depletion of H2A, H2B or H4 caused lethality. Scale bars, 500 μm.

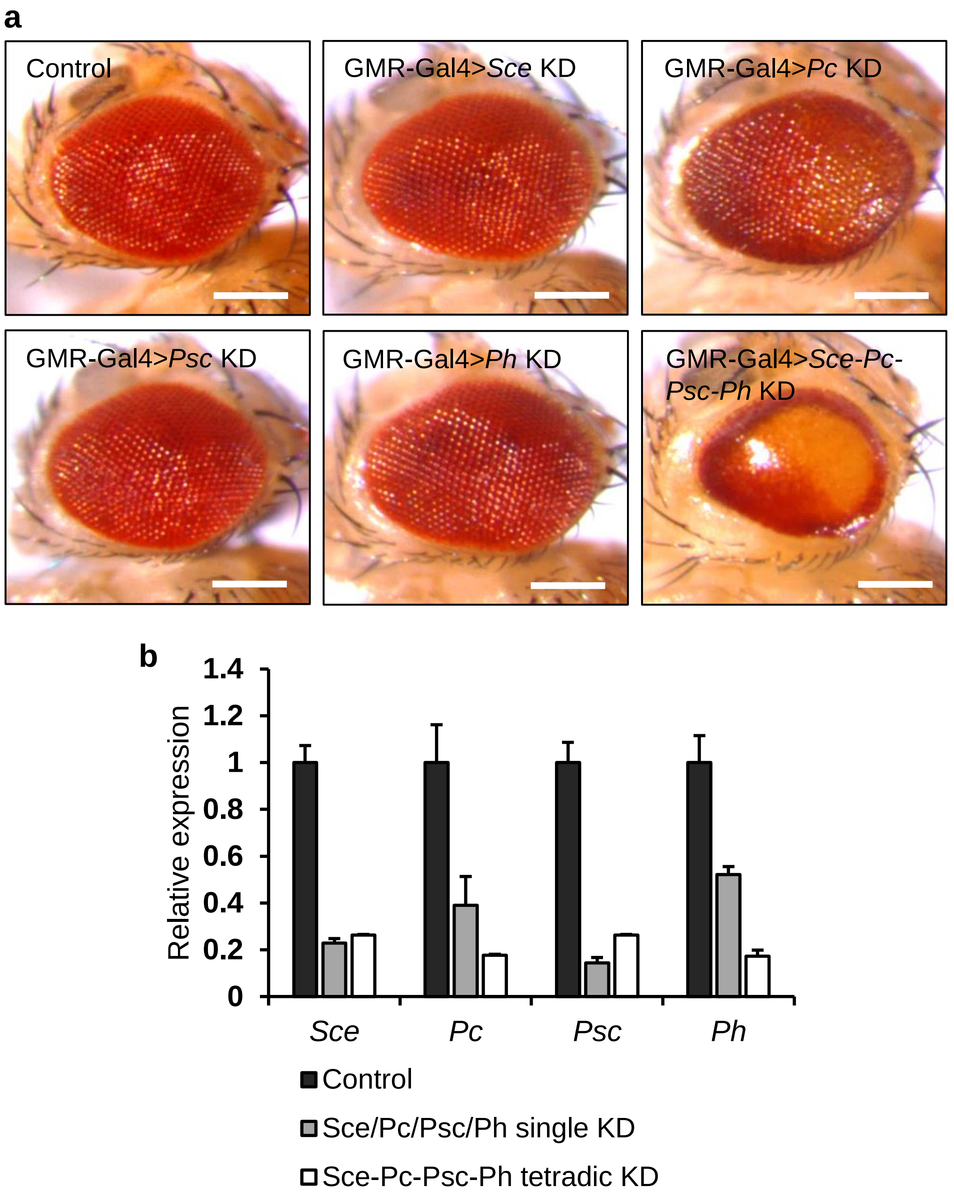

Supplementary Figure 6

Simultaneous knockdown of four subunits of PRC1 generated severe eye defects using the pNP system.

(a) Simultaneous knockdown of four subunits of PRC1 driven by GMR-Gal4 showed severe phenotypes: loss of pigmentation, fused ommatidia and small eyes, while knock down of one or two subunits of PRC1 exhibited no obvious phenotype. Scale bars, 200 μm. (b) qRT-PCR results show that the efficiency of single-gene knock down and tetradic knock down for *Sce, Pc, Psc, Ph* was comparable (n=3, mean±s.d.).

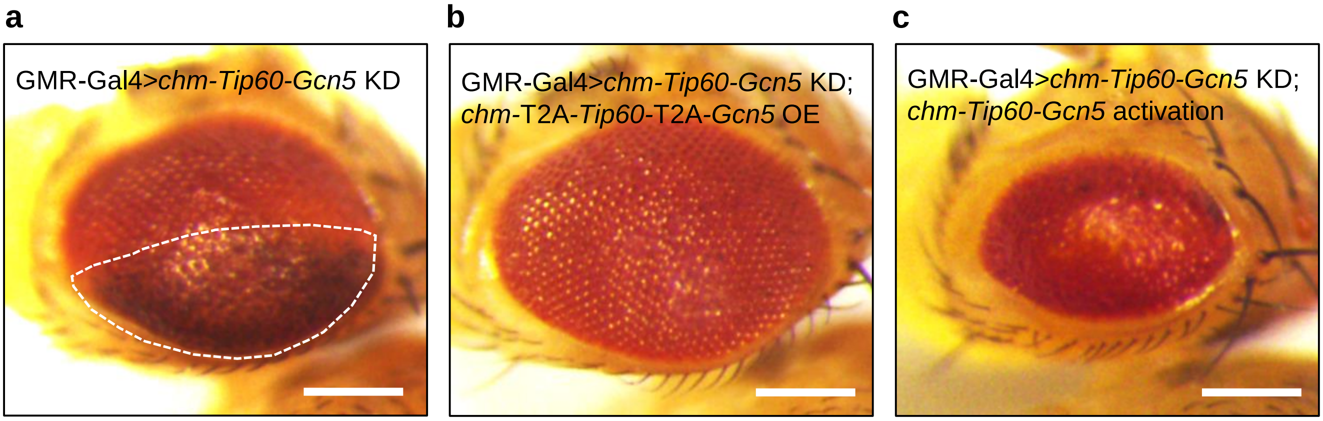

Supplementary Figure 7

Overexpression of *chm*-T2A-*Tip60*-T2A-*Gcn5* or activation of these HATs simultaneously can significantly rescue the eye defect phenotype in *chm-Tip60-Gcn5* KD flies.

Simultaneous knockdown of *chm-Tip60-Gcn5* using the pNP system driven by GMR-Gal4 produced severe eye defects (a), which can be rescued by overexpression of *chm*-T2A-*Tip60*-T2A-*Gcn5* (b) and the activation of *chm-Tip60-Gcn5* simultaneously (c). Scale bars, 200 μm.

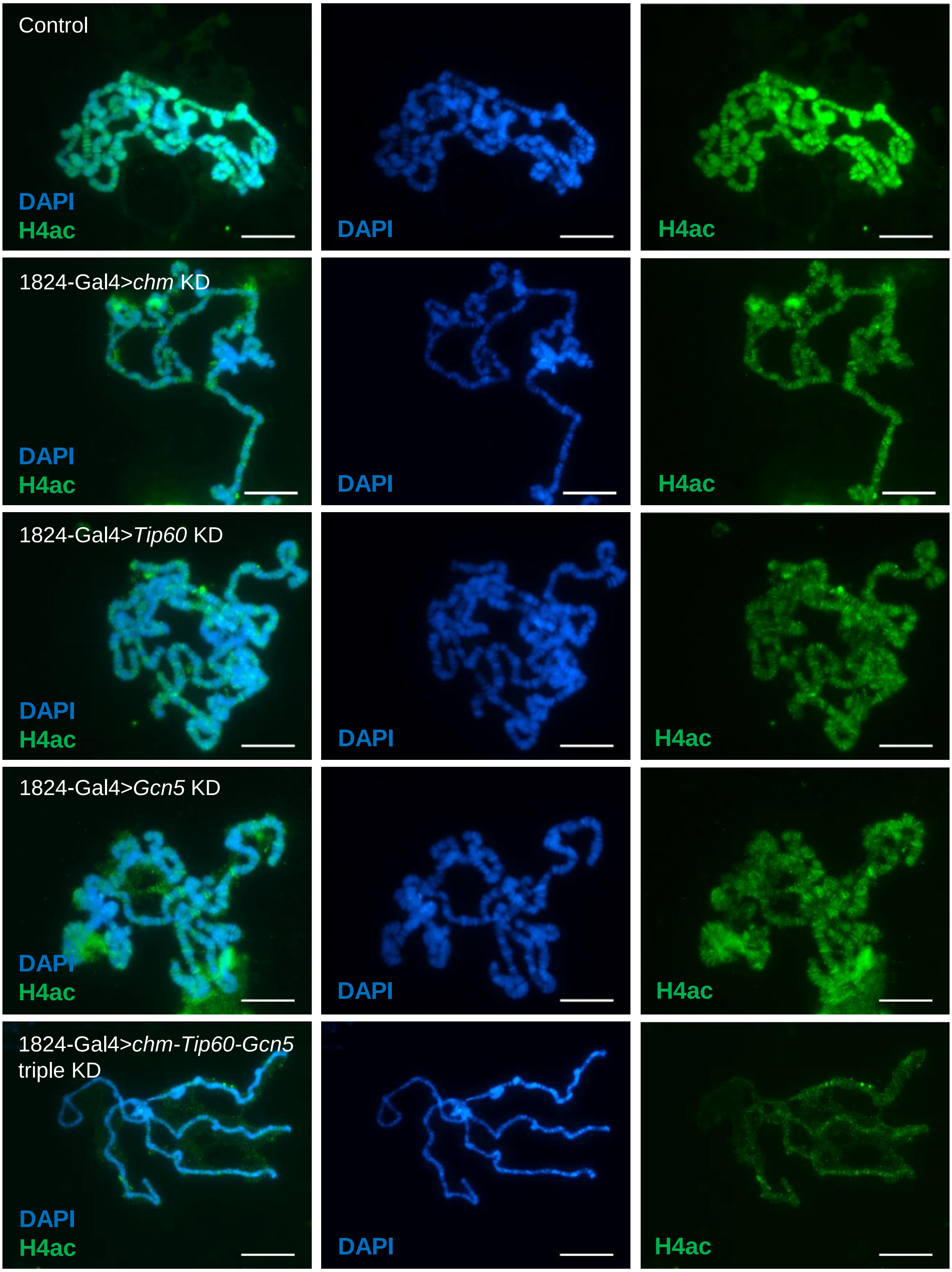

Supplementary Figure 8

Synergistic effects of HATs in histone acetylation.

Compared with control, single gene knockdown of *chm, Tip60, Gcn5* slightly decreased the global H4 acetylation level, while triple gene knockdown of *chm-Tip60-Gcn5* induced much less global H4 acetylation driven by 1824-Gal4. DNA was visualized with DAPI (blue) and H4ac was stained with rabbit polyclonal anti-acetyl-histone H4 antibody (green). Scale bars, 20 μm.

Supplementary Note 1: Sequence of pNP vector

CACCTAAATTGTAAGCGTTAATATTTTGTTAAAATTCGCGTTAAATTTTTGTTAAATCAGC
TCATTTTTTAACCAATAGGCCGAAATCGGCAAAATCCCTTATAAATCAAAAGAATAGAC
CGAGATAGGGTTGAGTGTTGTTCCAGTTTGGAACAAGAGTCCACTATTAAAGAACGT
GGACTCCAACGTCAAAGGGCGAAAAACCGTCTATCAGGGCGATGGCCCACTACGTG
AACCATCACCCTAATCAAGTTTTTTGGGGTCGAGGTGCCGTAAAGCACTAAATCGGAA
CCCTAAAGGGAGCCCCCGATTTAGAGCTTGACGGGGAAAGCCGGCGAACGTGGCG
AGAAAGGAAGGGAAGAAAGCGAAAGGAGCGGGCGCTAGGGCGCTGGCAAGTGTAG
CGGTCACGCTGCGCGTAACCACCACACCCGCCGCGCTTAATGCGCCGCTACAGGGC
GCGTCCCATTCGCCATTCAGGCTGCGCAACTGTTGGGAAGGGCGATCGGTGCGGGC
CTCTTCGCTATTACGCCAGCTGGCGAAAGGGGGATGTGCTGCAAGGCGATTAAGTTG
GGTAACGCCAGGGTTTTCCCAGTCACGACGTTGTAAAACGACGGCCAGTGAATTGTA
ATACGACTCACTATAGGGCGAATTGGGTACAAGCTTATTTATTTTGTTATGTTATATGTAT
TATATGTCAGACATAAAGAAAAGGAACACATCAAATGTGATAACAAAGACTAAACAAGT
AATTTTATTACACCAAAACGACAAAACAGTAGGCAGAACAAACAACGCATAGCCAAAC
ATTGACGAATTGGATACCCTGCCGATTGTCAGACACTTTTGTTGATCAGTTTCTTGCG
AATGGTCTCGTCCAGCGGTGGAATCGCCTCGCGGGGAATCAGAAAAGTGGACAGAT
TGAACAGATCCAGAAACACCTTGTACCGATCACTGAAACCAAAAAAAAACAAAGGGA
GAACAGTTTGAGTTCATTGATCCCCGATATAATCACATCTGCGATGATCACCTGAGAGT
GGAGCGCAGATATTGATAACCAGACGAGCCACCAGTGCCCAACTGTTGCGATCCAAT
CATGCGTTGCACCATGATCACGTGATTGTCTGCGGCGGGAATAGAAAGTATTTGGTTA
GGAAAACCAGTCTTAAACATAAGATATATTTATAAAAGAGTATCAAAGAATGCAATACTT
ACATCTCCACTTGGTTATTAACGAGTCGATGTCCATGAGCAGGGTGAGCAACTGGTG
TGGTTGGCTGAACCTGGGTTCATCCCTATAGAAGGTGATCATGATGGCTCCCTGAAG
GGCACGATGGCTAAACCGGCGATCCCCACGACGCACCAGTGCATCGTGCACTGCCG
GATCAAAGATGGAGCGATACACCTCGCGTCGCTTCTCAATGTCCATGAGGCGGTAGT
TTTTCGCCTTCTCCACGGGCTCCTCCATGGCGCTCTGTACCTGCGCCTCCAGGAATC
GATCGACGCTCTCCTGAAACTTGGCCCAGAAGTTGAAGCCACTCTCCTCCAGTCCG
GGCGTCCTCTCCAGCCATCGCTGCACTAGCTCCAGTAGCGAGGGATCTTTCTCCGA
GTTGCGAATCGAGTTCCGCGCCTCCTCGTCGCTAAAGACATCCGAGTACTTCTGGTT
GTATCTCACCCGCTGCTCTGTCAGAACTCCCAGCTTGTTCTCGATCAAACGGAACTG
CAGCGACTGAAAACCAGATGCGGGTGCCAGGTACTTGCGGAAGTCCATGAAGTCTA
GCGGGGTCATGGTCTCCAGAATGGGCACTTGGTCCACCAGGAGCTGTACAAAGGAA
GTTATAAACGGATTTTGGTAAGAGATTCAGAAAGCACTCACTTTTAGAATCAGAACCAC
TCGGTTCAGTCGCTTGACAATCTCCAGCGTCTTGGTTTCATCGATGACCTCTGCATCC
AACATGTCTCGTATGGAGTCGAACTCAAAGATGATCTGCTTGAACCAAAGCTCGTAGG
CTGTGGCGAAGGTACTTAAATGCCATTGAGTGTTGTCATCAAAGTTGTAAACCTACTC
ACCCTGGTGCGTGATGATGAACAGATGCTCATCGTGCACGGGTCGCTTGTCCTCCTC
GGACAGCATACACTGGGCATCCAGCAGTTTGTCCAGCATCAGATACTCTCCATAGATT

TTGCCCACTTCCGTGGTTAATGGCACCGCCGAATCATCGTGATCGTTTCTGTATGGGT
TTGAATTGAATCGCAGAACTGAAGATCGATTGGCATTCCTGGACAGCACGTGCTGGT
GCTCACCCGTTTCCTGCATAGGGACAGCTCATGGTGCACAGCTCAGATCAGATCGTG
ACTCCTCGACCGGCGGATGCTGGCGAACTGATCTCCGCCAGCGGACCGGAGATGA
GACCCCAGCGAACCGATAACAGAGCGAGAGAGCTCCAGTTCCGACTGATTGCACAG
TCGGTGATCTGGGCGATGGGCACTGCCAGATAGGCTGGGAATTATCAATCACTTGAG
GTGAAAGTGCGGCGCACACAAATCCAAGCTTGATATCATCGATCTCGACGCTGCATC
CAACGCGTTGGGAGCTCTCCGGATCAATTCGGCTTCACGTACCGTCGACGATGTAGG
TCACGGTCTCGAAGCCGCGGTGCGGGTGCCAGGGCGTGCCCTTGGGCTCCCCGG
GCGCGTACTCCACCTCACCCATCTGGTCCATCATGATGAACGGGTCGAGGTGGCGG
TAGTTGATCCCGGCGAACGCGCGGCGCACCGGGAAGCCCTCGCCCTCGAAACCGC
TGGGCGCGGTGGTCACGGTGAGCACGGGACGTGCGACGGCGTCGGCTGGTGCGG
ATACGCGGGGCAGCGTCAGCGGGTTCTCGACGGTCACGGCGGGCATGTCGACAAG
CCGAATTGATCCACTAGAAGGCCTAATTCGGTACACTATGCTAGTATAACTTCGTATAAT
GTATGCTATACGAAGTTATGCTAGTTGGCCACGTAATAAGTGTGCGTTGAATTTATTCG
CAAAAACATTGCATATTTTCGGCAAAGTAAAATTTTGTTGCATACCTTATCAAAAAATAA
GTGCTGCATACTTTTTAGAGAAACCAAATAATTTTTTATTGCATACCCGTTTTTAATAAAA
TACATTGCATACCCTCTTTTAATAAAAAATATTGCATACTTTGACGAAACAAATTTTCGTT
GCATACCCAATAAAAGATTATTATATTGCATACCCGTTTTTAATAAAATACATTGCATACC
CTCTTTTAATAAAGAATATTGCATACGTTGACGAAACAAATTTTCGTTGCATACCCAATA
AAAGATTATTATATTGCATACCTTTTCTTGCCATACCATTTAGCCGATCAATTCTGCTCG
GCAACAGTATATTTGTGGTGTGCCAACCAACAACctagcATAACTTCGTATAATGTATGC
TATACGAAGTTATGagctCGCTCGGGTAATCGCTTATCCTCGGGTAATCGCTTATCCTTA
AGCTGCAGGTCGGAGTACTGTCCTCCGAGCGGAGTACTGTCCTCCGAGCGGAGTAC
TGTCCTCCGAGCGGAGTACTGTCCTCCGAGCGGAGTACTGTCCTCCGAGCGGAGAC
TCCCGCGGTCGGAGTACTGTCCTCCGAGCGGAGTACTGTCCTCCGAGCGGAGTACT
GTCCTCCGAGCGGAGTACTGTCCTCCGAGCGGAGTACTGTCCTCCGAGCGGAGACT
CGTCGACGAGCTCGCCCGGGGATCGAGCGCAGCGGTATAAAAGGGCGCGGGGTGG
CTGAGAGCATCAGTTGTGAATGAATGTTCGAGCCGAGCAGACGTGCCGCTGCCTTC
GTTAATATCCTTTGAATAAGCCAACTTTGAATCACAAGACGCATACCAAAGTCTAGAAA
ACATCCCATAAAACATCCCATATTCAGCCGCTAGCATGGATGTTTTCCCAGTCACGAC
GTTGTAAAACGACGGCCAGTCTTAAGCTCGGGCCCCAAATAATGATTTTATTTTGACT
GATAGTGACCTGTTCGTTGCAACAAATTGATGAGCAATGCTTTTTTATAATGCCAACTT
TGTACAAAAAAGCAGGCTCCGCGGCCGCCCCCTTCACCGCTAGAGGAGAGCAACTG
CATAAGGCTATGAAGAGATACGCCCTGGTTCCTGGAACAATTGCTTTTACAGATGCAC
ATATCGAGGTGAACATCACGTACGCGGAATACTTCGAAATGTCCGTTCGGTTGGCAG
AAGCTATGAAACGATATGGGCTGAATACAAATCACAGAATCGTCGTATGCAGTGAAAA
CTCTCTTCAATTCTTTATGCCGGTGTTGGGCGCGTTATTTATCGGAGTTGCAGT

TGCGCCCGCGAACGACATTTATAATGAACGTGAATTGCTCAACAGTATGAACATTTCG
CAGCCTACCGTAGTGTTTGTTTCCAAAAAGGGGTTGCAAAAAATTTTGAACGTGCAAA
AAAAATTACCAATAATCCAGAAAATTATTATCATGGATTCTAAAACGGATTACCAGGGAT
TTCAGTCGATGTGAATTCAGGCGAGACATCGGAGTTGAAACTAAAACTGAATTACTAG
AGTGGACATATGCAcctagaCGCGATGCTCAAGGCAAAAAAAAATCAATCAAAAAACAAT
TTGAAAATTCCAAAAAAAAAGCATAATTGAACACAAAAAGTATAAAAACGGTAACAAGT
GTCTGATTTTGTTTATATCATTACATGAGCATGAAAACCAAAATTGAAAATTCTGAACTC
CCGCAGTCAAGTATCCCGAAACCTCAATCAATAAAACTATATTGGCGCATTCATGCTGC
CTAGGACTAGAGCAAACTAGTAGGATCCACTCGAGAAGATCTAGGTACCAGCGGCCG
CATCTAGTTCTGATCTGCTAGACAATTGTTGGCATCAGGTAGGCATCACACACGATTAA
CAACCCCTAAAAATACACTTTGAAAATATTGAAAATATGTTTTTGTATACATTTTTGATATT
TTCAAATAATACGCAGTTATAAAACTCATTAGCTAACCCATTTTTTCTTTGCTTATGCTTA
CAGATTGCAAAGAACTAGAGCCGCGGGATCTTTGTGAAGGAACCTTACTTCTGTGGT
GTGACATAATTGGACAAACTACCTACAGAGATTTAAAGCTCTAAGGTAAATATAAAATTT
TTAAGTGTATAATGTGTTAAACTACTGATTCTAATTGTTTGTGTATTTTAGATTCCAACCT
ATGGAACTGATGAATGGGAGCAGTGGTGGAATGCCTTTAATGAGGAAAACCTGTTTTG
CTCAGAAGAAATGCCATCTAGTGATGATGAGGCTACTGCTGACTCTCAACATTCTACTC
CTCCAAAAAAGAAGAGAAAGGTAGAAGACCCCAAGGACTTTCCTTCAGAATTGCTAAG
TTTTTTGAGTCATGCTGTGTTTAGTAATAGAACTCTTGCTTGCTTTGCTATTTACACCAC
AAAGGAAAAAGCTGCACTGCTATACAAGAAAATTATGGAAAAATATTTGATGTATAGTGC
CTTGACTAGAGATCATAATCAGCCATACCACATTTGTAGAGGTTTTACTTGCTTTAAAAA
ACCTCCCACACCTCCCCCTGAACCTGAAACATAAAATGAATGGAATTGTTGTTGTTAAC
TTGTTTATTGCAGCTTATAATGGTTACAAATAAAGCAATAGCATCACAAATTTCACAAATA
AAGCATTTTTTTCACTGCATTCTAGTTGTGGTTTGTCCAAACTCATCAATGTATCTTATC
ATGTCTGGTTCCAGAGCTCTGGCCACGTAATAAGTGTGCGTTGAATTTATTCGCAAAA
ACATTGCATATTTTCGGCAAAGTAAAATTTTGTTGCATACCTTATCAAAAAATAAGTGCT
GCATACTTTTTAGAGAAACCAAATAATTTTTTATTGCATACCCGTTTTTAATAAAATACATT
GCATACCCTCTTTTAATAAAAAATATTGCATACTTTGACGAAACAAATTTTCGTTGCATAC
CCAATAAAAGATTATTATATTGCATACCCGTTTTTAATAAAATACATTGCATACCCTCTTTT
AATAAAGAATATTGCATACGTTGACGAAACAAATTTTCGTTGCATACCCAATAAAAGATT
ATTATATTGCATACCTTTTCTTGCCATACCATTTAGCCGATCAATTCTGCTCGGCAACAG
TATATTTGTGGTGTGCCAACCAACAACGAGCTCCAGCTTTTGTTCCCTTTAGTGAGGG
TTAATTTCGAGCTTGGCGTAATCATGGTCATAGCTGTTTCCTGTGTGAAATTGTTATCC
GCTCACAATTCCACACAACATACGAGCCGGAAGCATAAAGTGTAAAGCCTGGGGTGC
CTAATGAGTGAGCTAACTCACATTAATTGCGTTGCGCTCACTGCCCGCTTTCCAGTCG
GGAAACCTGTCGTGCCAGCTGCATTAATGAATCGGCCAACGCGCGGGGAGAGGCGG
TTTGCGTATTGGGCGCTCTTCCGCTTCCTCGCTCACTGACTCGCTGCGCTCGGTCGT
TCGGCTGCGGCGAGCGGTATCAGCTCACTCAAAGGCGGTAATACGGTTATCCACAGA

ATCAGGGGATAACGCAGGAAAGAACATGTGAGCAAAAGGCCAGCAAAAGGCCAGGA
ACCGTAAAAAGGCCGCGTTGCTGGCGTTTTTCCATAGGCTCCGCCCCCCTGACGAGC
ATCACAAAAATCGACGCTCAAGTCAGAGGTGGCGAAACCCGACAGGACTATAAAGATA
CCAGGCGTTTCCCCCTGGAAGCTCCCTCGTGCGCTCTCCTGTTCCGACCCTGCCGC
TTACCGGATACCTGTCCGCCTTTCTCCCTTCGGGAAGCGTGGCGCTTTCTCATAGCTC
ACGCTGTAGGTATCTCAGTTCGGTGTAGGTCGTTCGCTCCAAGCTGGGCTGTGTGCA
CGAACCCCCCGTTCAGCCCGACCGCTGCGCCTTATCCGGTAACTATCGTCTTGAGTC
CAACCCGGTAAGACACGACTTATCGCCACTGGCAGCAGCCACTGGTAACAGGATTAG
CAGAGCGAGGTATGTAGGCGGTGCTACAGAGTTCTTGAAGTGGTGGCCTAACTACGG
CTACACTAGAAGGACAGTATTTGGTATCTGCGCTCTGCTGAAGCCAGTTACCTTCGGA
AAAAGAGTTGGTAGCTCTTGATCCGGCAAACAAACCACCGCTGGTAGCGGTGGTTTT
TTTGTTTGCAAGCAGCAGATTACGCGCAGAAAAAAAGGATCTCAAGAAGATCCTTTGA
TCTTTTCTACGGGGTCTGACGCTCAGTGGAACGAAAACTCACGTTAAGGGATTTTGGT
CATGAGATTATCAAAAAGGATCTTCACCTAGATCCTTTTAAATTAAAAATGAAGTTTTAAA
TCAATCTAAAGTATATATGAGTAAACTTGGTCTGACAGTTACCAATGCTTAATCAGTGAG
GCACCTATCTCAGCGATCTGTCTATTTCGTTCATCCATAGTTGCCTGACTCCCCGTCGT
GTAGATAACTACGATACGGGAGGGCTTACCATCTGGCCCCAGTGCTGCAATGATACCG
CGAGACCCACGCTCACCGGCTCCAGATTTATCAGCAATAAACCAGCCAGCCGGAAGG
GCCGAGCGCAGAAGTGGTCCTGCAACTTTATCCGCCTCCATCCAGTCTATTAATTGTT
GCCGGGAAGCTAGAGTAAGTAGTTCGCCAGTTAATAGTTTGCGCAACGTTGTTGCCAT
TGCTACAGGCATCGTGGTGTCACGCTCGTCGTTTGGTATGGCTTCATTCAGCTCCGG
TTCCCAACGATCAAGGCGAGTTACATGATCCCCCATGTTGTGCAAAAAAGCGGTTAGC
TCCTTCGGTCCTCCGATCGTTGTCAGAAGTAAGTTGGCCGCAGTGTTATCACTCATGG
TTATGGCAGCACTGCATAATTCTCTTACTGTCATGCCATCCGTAAGATGCTTTTCTGTG
ACTGGTGAGTACTCAACCAAGTCATTCTGAGAATAGTGTATGCGGCGACCGAGTTGCT
CTTGCCCGGCGTCAATACGGGATAATACCGCGCCACATAGCAGAACTTTAAAAGTGCT
CATCATTGGAAAACGTTCTTCGGGGCGAAAACTCTCAAGGATCTTACCGCTGTTGAGA
TCCAGTTCGATGTAACCCACTCGTGCACCCAACTGATCTTCAGCATCTTTTACTTTCAC
CAGCGTTTCTGGGTGAGCAAAAACAGGAAGGCAAAATGCCGCAAAAAAGGGAATAAG
GGCGACACGGAAATGTTGAATACTCATACTCTTCCTTTTTCAATATTATTGAAGCATTTA
TCAGGGTTATTGTCTCATGAGCGGATACATATTTGAATGTATTTAGAAAAATAAACAAAT
AGGGGTTCCGCGCACATTTCCCCGAAAAGTGC
